# INIM: Inertial Images Construction with Applications to Activity Recognition

**DOI:** 10.3390/s21144787

**Published:** 2021-07-13

**Authors:** Nati Daniel, Itzik Klein

**Affiliations:** 1 Technion-Israel Institute of Technology, 1st Efron st., Haifa 3525433, Israel; 2 Department of Marine Technologies, University of Haifa, 199 Aba Khoushy Ave., Haifa 3498838, Israel; kitzik@univ.haifa.ac.il

**Keywords:** activity recognition, two dimensional convolutional neural network, accelerometers, gyroscopes

## Abstract

Human activity recognition aims to classify the user activity in various applications like healthcare, gesture recognition and indoor navigation. In the latter, smartphone location recognition is gaining more attention as it enhances indoor positioning accuracy. Commonly the smartphone’s inertial sensor readings are used as input to a machine learning algorithm which performs the classification. There are several approaches to tackle such a task: feature based approaches, one dimensional deep learning algorithms, and two dimensional deep learning architectures. When using deep learning approaches, feature engineering is redundant. In addition, while utilizing two-dimensional deep learning approaches enables to utilize methods from the well-established computer vision domain. In this paper, a framework for smartphone location and human activity recognition, based on the smartphone’s inertial sensors, is proposed. The contributions of this work are a novel time series encoding approach, from inertial signals to inertial images, and transfer learning from computer vision domain to the inertial sensors classification problem. Four different datasets are employed to show the benefits of using the proposed approach. In addition, as the proposed framework performs classification on inertial sensors readings, it can be applied for other classification tasks using inertial data. It can also be adopted to handle other types of sensory data collected for a classification task.

## 1. Introduction

Human activity recognition (HAR) aims to classify the user activity in various applications such as gesture recognition [1,2], healthcare [3], home behaviour analysis [4], indoor navigation [5,6], and many more. Recently, several comprehensive survey papers were published, providing excellent review on applications and approaches to HAR [7,8,9,10,11].

Focusing on activity recognition for navigation applications, one of the branches of HAR is smartphone location recognition (SLR). For example, Pocket mode refers to the situation when the smartphone is placed in the user trousers and Swing mode refers to the case where the smartphone is held in the user hand while walking. The SLR goal is to classify the current location of the smartphone on the user. Commonly, both HAR and SLR utilizes the smartphone inertial sensors, namely the accelerometers and gyroscopes, readings to perform the classification task. Both HAR and SLR are gaining more attention in the navigation community. Applying activity recognition in traditional pedestrian dead reckoning (PDR) manged to improve the positioning accuracy [12,13,14,15,16]. In most traditional PDR algorithms the user step length is determined using an empirical formula. There, a re-calibrated gain is used in the process. This gain is very sensitive to the user dynamics and smartphone location. Using HAR and SLR algorithms user mode and smartphone locations are identified and their corresponding gain value can be used in the PDR step estimation process. Besides PDR, SLR was also shown to improve the performance of other navigation-related problems such as step length estimation [17,18,19] and adaptive attitude and heading reference system (AHRS) [20].

Currently, there are three major approaches to tackle HAR or SLR problems:

- Feature Based: features are extracted from the raw signals of the inertial sensors and used in classical machine learning algorithms.
- One Dimensional Deep Learning (1D-DL): the raw inertial sensor signals are plugged into one dimensional networks.
- Two Dimensional Deep Learning (2D-DL): the raw inertial sensors are transformed into two dimensional images and used as input for a network with the same dimensions.

Most of the approaches in the literature are focused on feature based and on 1D-DL networks. As such, there is no need to apply any 1D-2D transformation on the raw data. However, when using 2D-DL networks, the 1D inertial signals are first transformed into 2D space. Thus, compared to 1D-DL, an additional block is required in the algorithm. On the other hand, working with 2D-DL allows the implementation of strong proved architectures and tools derived in the computer vision field.

In 2D-DL, besides network architecture and hyper-parameter tuning as in 1D-DL, the major issue is how to transform the 1D inertial signal to a 2D image. The simplest approach is known as raw plots, where all relevant sensor outputs are plotted versus time and the result is used as an image for the 2D-DL classifier. For example, three axes accelerometer data were grouped by columns and the data collected from different positions are grouped by a row in the same image [21]. Unlike the raw plot method, the multichannel approach treats the same signals as a three overlapped color channels that correspond to red, green, and blue components in the RGB format by normalizing, scaling, and rounding a real value into an integer for pixel [21]. Recurrence plots are also used to create 2D images from sensors 1D signals. There, distance matrices capture temporal patterns in the signal and represent it as texture patterns in the image [22,23,24]. Another approach, is to construct an image using Fourier Transformation and create a spectrogram [25,26]. Gramian Angular Fields (GAF) and Markov Transition Fields (MTF) were applied to transform 1D time-series signals to 2D images [27,28]. Recently, an encoding technique for transforming an inertial sensor signal into an image with minimum distortion for image-based activity classification, known as Iss2Image, was proposed [29]. There, real number values from the accelerometer readings are transformed into three color channels to precisely infer correlations among successive sensor signal values in three different dimensions. In [29], Iss2Image approach was compared to other approaches and obtained state-of-the-art performance.

In this paper, an Inertial Image (INIM) framework for inertial based, smartphone location and human activity classification is derived. Here, the inertial signals, each represented as a one dimensional vector, are transformed into two dimensional matrices for the classification task. The motivation for this transformation is the ability to utilize strong proved architectures and tools derived in the computer vision field.

The contributions of the proposed framework are:

1. **Encoding**. A novel time series encoding approach based on accelerometers and gyroscopes readings. The three-axes accelerometers and three axes gyroscopes signals are encoded into a single RGB image.
2. **Transfer Learning**. To initialize the backbone deep-learning architecture, transfer-learning is applied form a residual network trained on the ImageNet [30] dataset. The dataset contains one thousand different labels and commonly used in computer vision domain. That is, the proposed transfer learning is performed between the computer vision domain to the inertial sensor domain.

To evaluate the proposed approach four different datasets are employed. Those contain 13 different labels of commonly used smartphone locations and human activities. Performance is compared relative to the original Iss2Image approach and also to an extension of this approach that enables taking the gyroscopes measurements for the encoding process. The results show that the proposed approach outperformed the other approaches on the examined dataset.

In addition, as the proposed framework performs classification on inertial sensors measurements, it can be applied for other classification tasks handling inertial data. It can also be adopted to handle other types of sensory data collected for a classification task.

The rest of the paper is organized as follows: Section 2 presents the leading approach for 2D classification using accelerometers data. Section 3 presents the proposed inertial images encoding and framework for inertial data based classification. Section 4 gives the experiential results on four different datasets and Section 5 presents the conclusions of this study.

## 2. Related Work Formulation—Iss2Image

The Iss2Image [29] approach is described in this section. It transforms accelerometer signals into colored images with minimum distortion and produces detailed correlations among successive accelerometer signals.

To describe the 1D-2D transformation, consider an activity sample *D*, including *N* accelerometer samples, each in three axes [x,y,z]:

(1) $$D=\left[\begin{array}{ccc} x_{1} & y_{1} & z_{1}\\ \vdots & \vdots & \vdots \\ x_{N} & y_{N} & z_{N} \end{array}\right]=\left[\begin{array}{ccc} X & Y & Z \end{array}\right]$$

The Iss2Image encoding technique has three steps:

- **Step 1**: Normalize all accelerometer signals and scale to 255, as follows:
  (2) $$\bar{x}=\frac{x-\min(X)}{\max(X)-\min(X)}\times 255$$
  (3) $$\bar{y}=\frac{y-\min(Y)}{\max(Y)-\min(Y)}\times 255$$
  (4) $$\bar{z}=\frac{z-\min(Z)}{\max(Z)-\min(Z)}\times 255$$
- **Step 2**: Convert the normalized accelerometer signals (2)–(4) into three integers corresponding to pixel values in the R,G,B channels of a color image, wherein each accelerometer signal value is treated as a pixel.
  For each sample of [x,y,z], using (2)–(4), three pixels are produced as follows:
  (5) $$R_{\bar{x}}=\left\lfloor \bar{x}\right\rfloor$$
  (6) $$G_{\bar{x}}=\left\lfloor \left(\bar{x}-\left\lfloor \bar{x}\right\rfloor \right)\times 10^{2}\right\rfloor$$
  (7) $$B_{\bar{x}}=\left\lfloor \left(\bar{x}\times 10^{2}-\left\lfloor \bar{x}\times 10^{2}\right\rfloor \right)\times 10^{2}\right\rfloor$$
  where ⌊x⌋ is the floor function, which takes the largest integer less than or equal to x∈R.
- **Step 3**: Generate and write a color image I=[RGB] using (5)–(7) as follows:
  (8) $$R=\left[\begin{array}{ccc} R_{{\bar{x}}_{1}} & R_{{\bar{y}}_{1}} & R_{{\bar{z}}_{1}}\\ \vdots & \vdots & \vdots \\ R_{{\bar{x}}_{N}} & R_{{\bar{y}}_{N}} & R_{{\bar{z}}_{N}} \end{array}\right]$$
  (9) $$G=\left[\begin{array}{ccc} G_{{\bar{x}}_{1}} & G_{{\bar{y}}_{1}} & G_{{\bar{z}}_{1}}\\ \vdots & \vdots & \vdots \\ G_{{\bar{x}}_{N}} & G_{{\bar{y}}_{N}} & G_{{\bar{z}}_{N}} \end{array}\right]$$
  (10) $$B=\left[\begin{array}{ccc} B_{{\bar{x}}_{1}} & B_{{\bar{y}}_{1}} & B_{{\bar{z}}_{1}}\\ \vdots & \vdots & \vdots \\ B_{{\bar{x}}_{N}} & B_{{\bar{y}}_{N}} & B_{{\bar{z}}_{N}} \end{array}\right]$$

Once the accelerometer signals were transformed to the color images, one can apply any 2D network to perform the classification task.

## 3. INIM: Inertial Images

**Figure 1:**
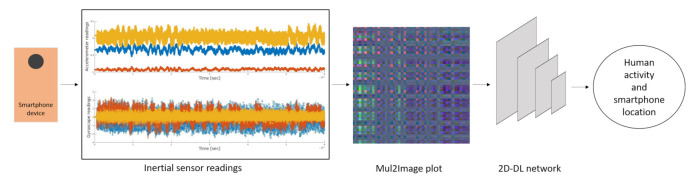
INIM framework: time series image coding with a backbone of a deep 2D-CNN used for inferring the smartphone location and human activity modes.

In this section, the proposed INIM framework is addressed. INIM is used for inferring the smartphone location or human activity based on a novel time-series coding method and a backbone 2D Deep Learning Network. INIM framework is illustrated in Figure 1. First, the smartphone’s accelerometer and gyroscope signals are collected. Then, the inertial sensor readings are transformed to a set of colored Red, Green, Blue (RGB) inertial images using the proposed encoding method, named Mul2Image. Then, these inertial images are divided into inertial patches (parts of the original inertial image) and are fed to the backbone 2D CNN network for the classification task.

### 3.1. Encoding Time-Series Signals to Inertial Images

The purpose of the Mul2Image encoder is to efficiently transform raw accelerometer and gyroscope signals into RGB inertial images. To that end, it is suggested to multiply the inertial sensor readings between themselves in the following manner: accelerometer x-axis readings are multiplied by gyroscopes x-axis readings, and the same for y and z axes. The motivation for such multiplication steams from the pattern recognition domain [31]. There, when the meta-data includes similarity properties of two time-series signals, the created image is similar in nature to either a sliding inner-product or the convolution operators of two functions.

**Figure 2:**
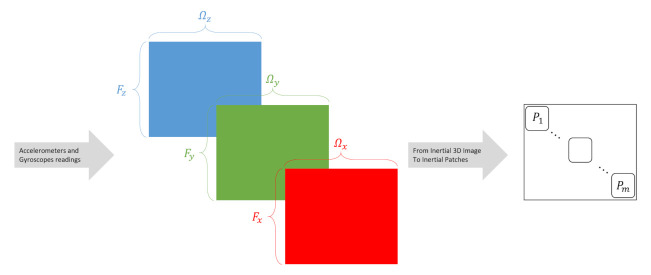
Illustration of the Mul2Image time-series encoding approach. It constructs inertial images (INIM) from inertial sensors readings.

The proposed encoding approach requires six steps as described below:

- **Step 1**: Normalize the specific force vector, f, (accelerometer output):
  (11) $$\hat{f}=\frac{f}{\left||f|\right|}$$
  where $\hat{f}$ is the normalized specific force vector defined at epoch (time index) *k* by its three components:
  (12) $${\hat{f}}_{k}={\left[\begin{array}{ccc} f_{k,x} & f_{k,y} & f_{k,z} \end{array}\right]}^{T}$$
- **Step 2**: The normalized accelerometer signals from *n* epochs are stacked in matrix $F\in R^{n\times 3}$:
  (13) $$F=\left[\begin{array}{ccc} f_{1,x} & f_{1,y} & f_{1,z}\\ \vdots & \vdots & \vdots \\ f_{n,x} & f_{n,y} & f_{n,z} \end{array}\right]=\left[\begin{array}{ccc} F_{x} & F_{y} & F_{z} \end{array}\right]$$
- **Step 3**: Each angular velocity measurement (gyroscope output) vector
  (14) $$\omega_{k}={\left[\begin{array}{ccc} \omega_{k,x} & \omega_{k,y} & \omega_{k,z} \end{array}\right]}^{T}$$
  from *n* samples are used to construct the following $\Omega \in R^{n\times 3}$ matrix:
  (15) $$\Omega =\left[\begin{array}{ccc} \omega_{1,x} & \omega_{1,y} & \omega_{1,z}\\ \vdots & \vdots & \vdots \\ \omega_{n,x} & \omega_{n,y} & \omega_{n,z} \end{array}\right]=\left[\begin{array}{ccc} \Omega_{x} & \Omega_{y} & \Omega_{z} \end{array}\right]$$
- **Step 4**: Using (13) and (15) the RGB layers are constructed:
  (16) $$R=F_{x}\cdot {\Omega_{x}}^{T}\in R^{n\times n}$$
  (17) $$G=F_{y}\cdot {\Omega_{y}}^{T}\in R^{n\times n}$$
  (18) $$B=F_{z}\cdot {\Omega_{z}}^{T}\in R^{n\times n}$$
  Then, the inertial image, termed INIM, is constructed stacking the three layers:
  (19) $$I=\left[R,G,B\right]\in R^{n\times n\times 3}$$
- **Step 5**: The values of (19) are scaled in the range of [0,255] by multiplying them by 255.
- **Step 6**: Finally, the image I is cropped into *m* non-overlapped patches $P_{1},\cdots ,P_{m}$ based on the network input size *s*, as follows:
  (20) $$m=\left\lfloor \frac{n}{s}\right\rfloor$$
  where $P_{k}$, k=1,2,…,m is of dimension s×s.
  Notice that the total number of patches is $\left\lfloor n^{2}/s^{2}\right\rfloor$, yet we require no overlap between the accelerometer and gyroscopes data. Therefore, only the diagonal patches are taken into account, resulting in $\left\lfloor n/s\right\rfloor$ patches, as illustrated in Figure 2.

To summarize, the output of the Mul2Image encoder consists of *m* non-overlapped patches, as shown in Figure 2. The size of the inertial patches is based on the number of samples *n* samples and the network input size *s*. The network input size is a configurable parameter determined by the user.

When comparing Mul2Image to Iss2Image it is observed that Iss2Image uses only accelerometer readings while Mul2Image uses both accelerometer and gyroscope measurements. In addition, Mul2Image creates a wider image which consists additional information and, thus, enriches the input of the network enabling it to perform better.

### 3.2. Backbone 2D Network Architectures

Over recent years, deep learning and, in particular, deep convolution neural networks (CNN) play a major role in computer vision applications. Unlike classical machine learning techniques, in deep learning the net performs representation learning which allows the machine to be fed with raw data and automatically discover the representations needed for the classification task. One of the major challenges in very deep CNN is coping with exploding gradients during the training procedure. To avoid such situations, residual network (ResNet) architecture uses skip connections to enable the gradients to flow easily from a CNN layer to the other. In that manner, the problem of network accuracy degradation is resolved.

Therefore, in this work, the ResNet50 [32] network is adopted as the feature extraction backbone and classification model for SLR and HAR tasks. As it name implies, ResNet50 uses 50 residual layers of network. Network initialization is done by a transfer learning method of a pre-trained network—ImageNet [30]. The weights of trained models on ImageNet database consists of about 1.2 million labeled images divided in 1000 different classes. The architecture of the backbone ResNet50 2D-CNN model is summarized in detail in Table 1.

### 3.3. Summary

**Figure 3:**
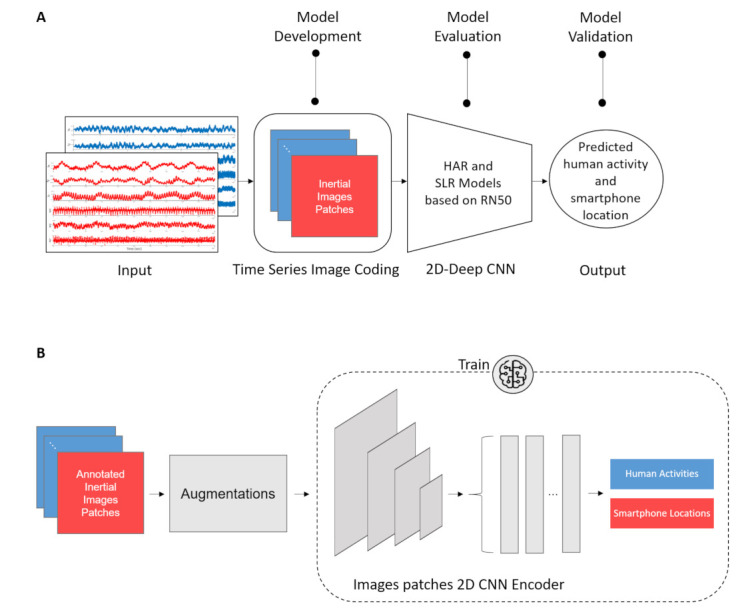
Proposed INIM framework for smartphone location and human activity recognition using the smartphone’s accelerometers and gyroscopes. (**A**) Main building blocks, including Mul2Image and ResNet50 as a backbone architecture. (**B**) End-to-end training procedure.

Figure 3 summarizes the INIM framework showing the main building blocks in Figure 3A and the end-to-end training procedure in Figure 3B.

The main building block includes the Mul2Image time series image coding algorithm (Section 3.1), the 2D backbone ResNet50 architecture based on transfer-learning (initialization) from weights trained on ImageNet (Section 3.2), and the entire process from the inertial sensors’ raw data to the classification result.

## 4. Analysis and Results

### 4.1. Datasets

Four different datasets are used for the evaluation of the proposed approach and comparison to other approaches. All those datasets contain accelerometers and gyroscopes measurements, each labeled with a specific activity label. Three datasets consist of HAR activities and one with SLR as follows:

- **HAR1** [33]. This dataset was recorded, with 50Hz sampling rate, by 10 people. Five sets of inertial sensors were placed in: right jeans pocket, left jeans pocket, belt, right upper arm, and right wrist. Seven human activities were considered: Biking, Stationary, Sitting, Downstairs, Upstairs, Walking, and Jogging.
- **HAR2** [34]. In this dataset 15 people (8 males/ 7 females) recorded inertial data sampled in 50Hz using seven sets of inertial sensors, each placed in a different location on the user: chest, forearm, head, shin, thigh, upper arm, and waist. There eight types of human activities were addressed: Stationary, Sitting, Downstairs, Upstairs, Walking, Jogging, Jumping, and Lying. In this work, only the first six activities were considered as they are most relevant to indoor navigation activities.
- **HAR3 and HAR4** [35]. This dataset was recorded using three sets of inertial sensors: on the chest, attached over the wrist on the dominant arm, and on the dominant side’s ankle. Nine people collected the data which were sampled at 100 Hz. The dataset contains 18 different user activities, some describing dynamics related human activities like in HAR1 and HAR2 and some describing working with home appliances activities. In this work the activities of Downstairs and Upstairs were chosen to construct the HAR3 dataset. HAR4 datasets, contains Ironing and Vacuum cleaning activities. In that manner, distinguish is made between the activities types and nature.
- **SLR** [36]. In this dataset, recordings were made during walking. Seven people, each with a different smartphone, recorded 190 minuets of inertial data in sampling rate between 25 and 100 Hz. There, four smartphone modes were addressed: Pocket, Texting, Swing, and Talking.

These datasets were chosen as they are commonly used for baseline benchmarking of HAR and SLR problems and all of them were constructed for evaluating deep-learning approaches. In HAR1, HAR2 and HAR3 focused was given to dynamics related human activities, The difference between the three datasets is the inertial sensor locations and their type, sampling rate and different people who made the recordings. The activity list of each dataset is summarized in Table 2 showing 13 different classes.

**Figure 4:**
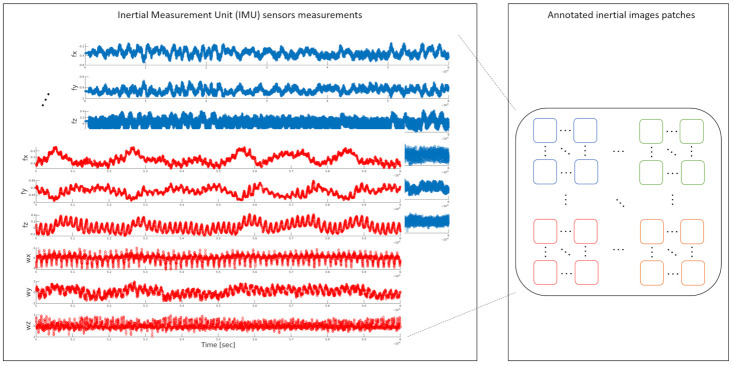
Illustration of inertial images construction. Each cluster of colored boxes indicates different images patches, each with a different activity, constructed from the raw inertial signals.

**Figure 5:**
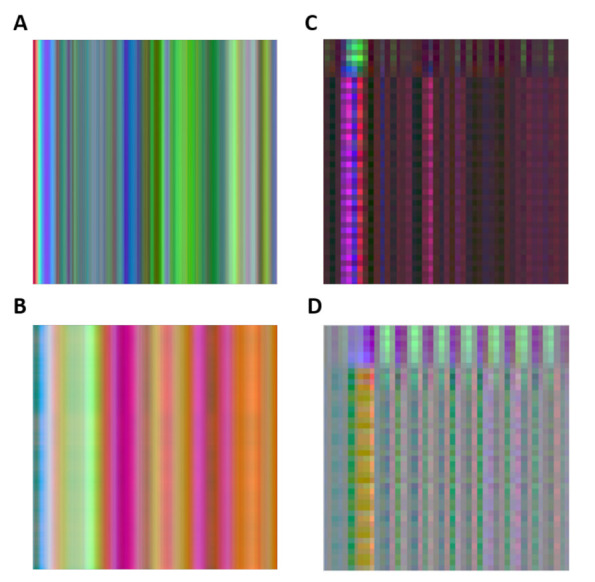
Illustration of inertial image patches as a function of different smartphone locations obtained from SLR dataset: (**A**) Pocket, (**B**) Texting, (**C**) Talking, and (**D**) Swing.

After choosing the datasets, the corresponding inertial images and their patches were constructed following the steps described in Section 3.1 and presented in Figure 4. An illustration of this procedure is presented in Figure 5 showing the raw inertial sensor signals (three accelerometers and three gyroscopes) and corresponding four inertial image and their patches, where each image represents a different class (more inertial patches are shown in Appendix A).

Note that each inertial image is divided into smaller patches based on the network input size as mentioned in Section 3, and labelled as the base inertial image category class.

The overall numbers, per activity and dataset are shown in Table 3 using a width size of 224 pixels. Figure 5 shows an example of inertial image patches for four different smartphone locations generated by the proposed Mul2Image encoder.

### 4.2. Experimental Setup

As presented in Table 3, a total of 26361 image patches constructed from raw inertial data and representing 13 different classes were constructed. To divide this dataset into train and test, in each class the images were randomly divided to 75% images for training and the other 25% of the images for testing.

For each dataset, as described in Table 2, a different ResNet50 deep convolutional neural networks was trained. There, the input image size was obtained in a similar manner. Cropping the full inertial image into patches of 224 × 224 pixels without overlapping. This resolution was chosen because it was shown to be the optimal size for ResNet50 model. Transfer learning, from ImageNet, was used to update the model, together with hyper-parameters fine-tuning. Thus, is, in current training of the new classes, ImageNet weight was used to initiate the training process. Three data augmentations, namely rotation, translation, and flipping were applied in the train phase. A comparison of the performance with and without data augmentation was made to verify the augmentation ability to improve the classification performance. Results on the HAR1 dataset, provided at the Appendix B, clearly show the improvement with data augmentation. As consequence, data augmentation has been applied to all the other datasets.

The model was trained with a mini-batch of size 24 and optimized using adaptive moment estimation (Adam) [37] algorithm, which computes the learning rates for each parameter during the training. An initial learning rate of λ=0.0001 with a discounting factor for the history/coming gradient of ρ=0.99, a learn rate drop factor of 0.5, and piecewise schedule of 5.

The network was run for 10 epochs, 148 iterations for training and 50 for validation. The model coding was implemented using Matlab, and trained on a single NVIDIA GeForce GTX 1080 GPU. This end-to-end process was repeated three times to check the stability of the results.

### 4.3. Encoders: Image Size and Computational Speed

The proposed approach is compared to Iss2Image solution, which is considered a state-of-the-art method in image construction from inertial measurements. The proposed approach uses both accelerometer and gyroscope measurements, while Iss2Image uses only the former. Thus, to make a fair comparison the Iss2Image approach was elaborated to include gyroscopes measurements as well. To that end, instead of working with an image patch with size 224 × 3 × 3 pixels, the extended Iss2Image (eIss2Image) now works with 224 × 6 × 3 pixels. In the proposed approach the image patch has the size of 224 × 224 × 3 pixels, that is approximately 37 times more pixels in the patch. As a consequence, the time required to create the image patches in the proposed approach is larger than the two versions of Iss2Image. For example, the time required to construct ten image patches for the Iss2Image is 0.25 s while for Mul2Image 0.39 s, that is about 64% faster than the proposed encoder. The image patches and average time to construct ten image patches for each approach are given in Table 4. Notice that since Iss2Image images have smaller size, they contain less pixel data-type information that may assist to the classification task.

### 4.4. SLR

SLR classification task is considered. For a fair comparison between the proposed Mul2Image encoder and the one used in Iss2Image, the same backbone network, that is ResNet50, was used for all the encoders. In addition, the 2D architectures are also compared to a 1D deep-learning CNN based network as used in [36].

Table 5 shows the average recognition training accuracy for the train dataset for each approach and smartphone location. All 2D approaches obtained better accuracy than the 1D-CNN approach.

As the test dataset is balanced and the accuracy of each class is high, Table 6 shows the total (average on all classes) test dataset accuracy. All the 2D-DL approaches achieved higher accuracy than the 1D-DL approach. In addition, the recognition results of all the 2D-DL approaches are impressive, and yielded high obtained an accuracy of more than 98% on the testing set. The suggested encoder and the modified eIss2Image obtained better performance than Iss2Image. Between the two, the former achieved the best performance with 99.7% accuracy.

### 4.5. HAR

The HAR classification task is considered using the four datasets with different classes as described in Table 2. As in the SLR, for a fair comparison between the proposed Mul2Image encoder and the one used in Iss2Image, the same backbone network, that is ResNet50, was used for all the encoders.

Table 7 shows the accuracy of the 2D-DL approaches for the train and test of HAR1 dataset. The overall test accuracy of the proposed encoder performs better than the other compared methods (Iss2Image and eIss2Image). For the Downstairs activity, Iss2Image obtained accuracy of 78.1% in the test dataset, while the proposed Mul2Image yielded accuracy of 88.0%, that is a 9.9% improvement. In the same manner, for the Upstairs activity, an improvement of 47.4% was achieved. Mul2Image also improved the accuracy of eIss2Image in Downstairs and Upstairs activities by 4.7% and 43.1%, respectively. In Stationary mode Mul2Image performed worse than the other approaches. The addition of gyroscopes measurements to the Iss2Image encoder (eIss2Image), shows better performance on 6 out of 7 classes than the encoder based only on accelerometers (Iss2Image).

In Table 8, the average recognition accuracy of the three encoders is given for HAR2 train and test dataset. The Mul2Image encoder shows best performance on all the six activities, with 100%, 94.0%, 96.0%, 98.6%, 95.6%, and 94.0% accuracy in the Jogging, Sitting, Downstairs, Upstairs, Stationary, and Walking classes, respectively. In particular, the ability to distinguish between Downstairs to other learned classes fails in Iss2Image and eIss2Image methods (24–26%), while in Mul2Image, an improvement of 70% was achieved. Besides the Downstairs class, Iss2Image achieved 17% accuracy in Stationary mode while 37.7% was obtained by eIss2Image in Walking mode. In both classes, Mul2Image got an accuracy of more than 94.0%.

Table 9 and Table 10 shows the train and test accuracy for HAR3 and HAR4 datasets using the three encoders. Using Table 9 a classification metric, called bias is analyzed. Bias Upstairs–Downstairs indicates how much bias one category has over the other when lower bias indicates a better classifier. As can be seen, the highest accuracy of 98.1%, was achieved when using eIss2Image encoder to Upstairs recognition. However, the bias is 17%, which means the network has high bias towards Upstairs. In Iss2Image, the bias is even higher and reaches 20.3%. On the other hand, Mul2Image encoder yields 91.7% recognition accuracy of predicting Upstairs class, but has a bias of only 9.9%.

Table 10, shows that Mul2Image yields overall higher recognition accuracy on both Ironing and VacuumCleaning classes than the compared methods. However, the Mul2Image bias VacuumCleaning - Ironing is higher by a factor of 2.8 and 3.9 towards Ironing, compared to eIss2Image and Iss2Image, respectively.

Comparing the bias metric of Mul2Image method in both Table 9 and Table 10, it is observed that user dynamic influences the bias value. When the dynamic is similar, like between Upstairs and Downstairs, the bias is smaller. On the other hand, in different dynamic types, like between Ironing and VacuumCleaning, the resulted bias was higher.

To summarize the results of Table 5, Table 6, Table 7, Table 8, Table 9 and Table 10, a weighted average accuracy on each of the test datasets and a corresponding final ranking of each encoder are calculated and presented in Table 11. The weighted average accuracy is calculated per number of patches in each activity (which are provided in Table 3). The final rank is the indicator of the highest weighted performance over the entire datasets. The proposed Mul2Image encoder obtained the best overall accuracy reaching 88.6%. Except of HAR3, using Mul2Image obtained also the best performance in all other datasets. Particularly, an improvement of 22.7% in HAR1 dataset and 8.8% in HAR4 dataset.

## 5. Conclusions

Human activity recognition is an important task in various applications like healthcare, gesture recognition and indoor navigation. In the latter, smartphone location recognition is gaining more attention as a critical operation as it enhances indoor positioning accuracy. In this paper, a framework for both human activity and smartphone location recognition, based on the smartphone’s inertial sensors, was proposed. This framework, termed INIM for inertial images, transforms the accelerometer and gyroscope signals into images enabling the usage of proved architectures and tools from the computer vision domain.

The main contributions of this work are a novel time series encoding approach, from inertial signals to inertial images, Mul2Image, and demonstrating transfer learning from computer vision domain (using ImageNet) to the inertial based classification of HAR and SLR problems.

To evaluate the proposed approach four different datasets, contain 13 different labels, were employed. Performance (in terms of accuracy) was compared relative to the original Iss2Image approach and also to an extension of this approach that the usage of gyroscopes measurements in the encoding process. To make a fair comparison between those three encoders, the same backbone ResNet50 was employed. In addition, the SLR task, performance was compared also to a leading 1D-CNN architecture. Results show that the proposed extension of the Iss2Image encoder obtained better performance than the original Iss2image approach. The proposed Mul2Image encoder, obtained the overall best accuracy of 88.6% improving Iss2image accuracy by 7.8% and eIss2image by 4.7%.

In addition, the INIM framework or its Mul2Image encoder, can be applied to any other classification tasks using inertial sensors data. Moreover, it can also be adopted to handle other types of sensory data collected for any type of a classification task.

## Figures and Tables

**Table 1:** ResNet50 architecture used as backbone within the INIM framework.

Layer Name	Output Size [Pixels]	50-Layer Structure [Kernel Size, # Channels]
conv1	112 × 112	stride 2, 7 × 7, 64
conv2	56 × 56	stride 2, max pool 3 × 3 $\left[\begin{array}{c} 1\times 1,64\\ 3\times 3,64\\ 1\times 1,256 \end{array}\right]$ X3
conv3	28 × 28	$\left[\begin{array}{c} 1\times 1,128\\ 3\times 3,128\\ 1\times 1,512 \end{array}\right]$ X4
conv4	14 × 14	$\left[\begin{array}{c} 1\times 1,256\\ 3\times 3,256\\ 1\times 1,1024 \end{array}\right]$ X6
conv5	7 × 7	$\left[\begin{array}{c} 1\times 1,512\\ 3\times 3,512\\ 1\times 1,2048 \end{array}\right]$ X3

**Table 2:** Activity list of each dataset.

SLR [36]	HAR1 [33]	HAR2 [34]	HAR3 [35]	HAR4 [35]
Pocket	Biking	Stationary	Downstairs	Ironing
Texting	Stationary	Sitting	Upstairs	VacuumCleaning
Swing	Sitting	Downstairs		
Talking	Downstairs	Upstairs		
	Upstairs	Walking		
	Walking	Jogging		
	Jogging			

**Table 3:** Number of train and test generated inertial Image patches (number of train, number of test) with a width size of 224 pixels for different activities and datasets.

Activity/Dataset	SLR	HAR1	HAR2	HAR3	HAR4
Pocket	1064, 355	-	-	-	-
Texting	1808, 603	-	-	-	-
Swing	441, 147	-	-	-	-
Talking	242, 81	-	-	-	-
Biking	-	932, 233	-	-	-
Stationary	-	932, 233	1144, 159	-	-
Downstairs	-	932, 233	411, 50	986, 418	-
Upstairs	-	699, 116	534, 70	1098, 470	-
Walking	-	932, 233	789, 167	-	-
Jogging	-	932, 233	472, 62	-	-
Sitting	-	932, 233	1299, 149	-	-
Ironing	-	-	-	-	2229, 963
VacuumCleaning	-	-	-	-	1649, 696

**Table 4:** Comparison of image patches construction time on different image encoding methods.

Image Encoder Method	Average Time [Sec] per Ten Image Patches	Size of Images Patches [Pixels]
Iss2Image	0.25	224 × 3 × 3
eIss2Image	0.26	224 × 6 × 3
Mul2Image (Ours)	0.39	224 × 224 × 3

**Table 5:** Average recognition training accuracy (%) on SLR dataset with ResNet50 2D-CNN architecture.

Smartphone Location	1D-CNN	Iss2Image	eIss2Image	Mul2Image (Ours)
Pocket	98.8%	98.9%	99.7%	99.2%
Texting	96.9%	99.2%	99.8%	99.8%
Swing	97.2%	98.0%	99.3%	100%
Talking	95.7%	97.5%	100%	96.3%

**Table 6:** Total recognition test accuracy (%) on the SLR dataset with ResNet50 2D-CNN architecture.

Method	Accuracy
1D-CNN	97.7%
Iss2Image	98.8%
eIss2Image	99.7%
Mul2Image (Ours)	99.1%

**Table 7:** Average recognition accuracy (%) on HAR1 train/test datasets for each activity.

Activity	Image Encoder Method		
	Iss2Image	eIss2Image	Mul2Image (Ours)
Jogging	99.6%/100%	100%/100%	99.6%/99.1%
Sitting	78.5%/65.7%	96.6%/91.0%	95.3%/97.0%
Downstairs	88.0%/78.1%	95.3%/83.3%	99.1%/88.0%
Upstairs	82.9%/45.7%	90.9%/50.0%	98.9%/93.1%
Stationary	90.1%/55.4%	97.9%/71.7%	80.3%/42.1%
Walking	90.6%/79.0%	97.0%/77.3%	98.7%/85.4%
Biking	97.0%/99.1%	100%/99.6%	94.8%/96.6%

**Table 8:** Average recognition accuracy (%) on HAR2 train/test datasets for each activity.

Activity	Image Encoder Method		
	Iss2Image	eIss2Image	Mul2Image (Ours)
Jogging	93.2%/100%	93.2%/100%	99.2%/100%
Sitting	99.7%/100%	100%/99.3%	95.7%/94.0%
Downstairs	68.2%/24.0%	71.8%/26.0%	96.4%/96.0%
Upstairs	74.4%/95.7%	78.9%/95.7%	97.0%/98.6%
Stationary	98.6%/17.0%	100%/79.2%	88.1%/95.6%
Walking	85.8%/76.0%	91.4%/37.7%	95.4%/94.0%

**Table 9:** Average recognition accuracy (%) on HAR3 train/test datasets for each activity.

Activity	Image Encoder Method		
	Iss2Image	eIss2Image	Mul2Image (Ours)
Downstairs	95.2%/77.0%	98.0%/81.1%	88.8%/81.8%
Upstairs	99.2%/97.3%	92.4%/98.1%	89.4%/91.7%

**Table 10:** Average recognition accuracy (%) on HAR4 train/test datasets for each activity.

Activity	Image Encoder Method		
	Iss2Image	eIss2Image	Mul2Image (Ours)
Ironing	99.0%/71.9%	98.0%/72.0%	87.5%/86.6%
VacuumCleaning	96.9%/74.5%	99.1%/75.6%	80.4%/76.4%

**Table 11:** Weighted average accuracy results using the three encoders with the same ResNet50 backbone. The final ranking is based on the weighted test accuracy (%).

Image Encoder Method	Dataset					Overall Accuracy	Final Rank
	SLR	HAR1	HAR2	HAR3	HAR4		
Iss2Image	98.8%	67.6%	76.9%	87.7%	72.9%	80.8%	3
eIss2Image	99.7%	72.9%	84.3%	90.0%	73.5%	83.9%	2
Mul2Image (Ours)	99.1%	95.6%	85.3%	88.6%	82.3%	88.6%	1

**Figure A1:**
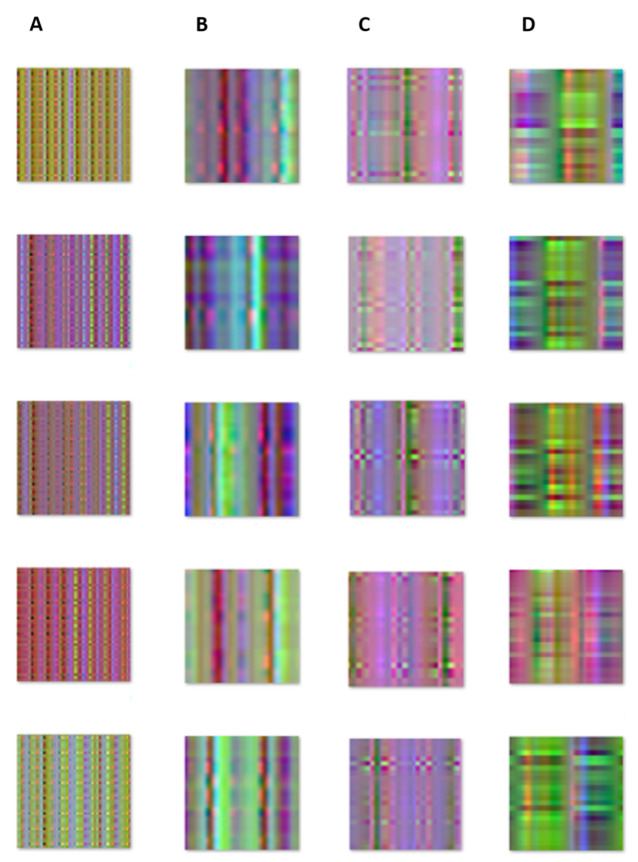
Batch illustration of inertial image patches as a function of the smartphone locations obtained from SLR dataset: (**A**) Pocket, (**B**) Texting, (**C**) Talking, and (**D**) Swing.

## Appendix A

Figure A1 shows a batch illustration of inertial image patches as a function of the smartphone locations on the SLR dataset: (A) Pocket, (B) Texting, (C) Talking, and (D) Swing. Images of size 224 pixels as generated by the Mul2Image encoder.

## Appendix B

Table A1 shows the accuracy results with and without data augmentation for the HAR1 dataset with the proposed Mul2Image encoder. Bias with and without data augmentation indicates how much bias one category has over the other when lower bias indicates a better classifier. The overall bias is 24.6% and indicates that using data augmentation in the training procedure improves the final test classification performance.

**Table A1:** Average recognition accuracy (%) on HAR1 train/test datasets for each activity with and without data augmentation.

Activity	Mul2Image (Ours)	
	With Data Augmentation	Without Data Augmentation
Jogging	99.6%/99.1%	98.2%/88.4%
Sitting	95.3%/97.0%	92.9%/94.8%
Downstairs	99.1%/88.0%	98.9%/90.1%
Upstairs	98.9%/93.1%	98.1%/73.3%
Stationary	80.3%/42.1%	79.6%/66.5%
Walking	98.7%/85.4%	96.1%/85.8%
Biking	94.8%/96.6%	82.5%/77.7%
